# Impact of Bone-Borne and Miniscrew-Assisted Rapid Palatal Expansion on Airway Dimensions and Postexpansion Stability: Evidence From Cone-Beam CT Studies

**DOI:** 10.7759/cureus.98578

**Published:** 2025-12-06

**Authors:** Nada N Al-Madani, Lamees A Al Salamah, Mohammed H Almutairi, Ali A Alaithan, Abdulrahman H Alsaedi, Reem A Aloraini, Joud A Aljabr, Ghadah A AlGhamdi, Abrar M Alrubayan, Latifah M Alfrihidi, Feras Y Dahhas, Ahmad A Baqer

**Affiliations:** 1 Orthodontics and Dentofacial Orthopaedics, Security Forces Hospital, Khobar, SAU; 2 General Dentistry, Princess Nourah Bint Abdulrahman University, Riyadh, SAU; 3 General Dentistry, Ministry of National Guard - Health Affairs, Riyadh, SAU; 4 College of Dentistry, Imam Abdulrahman Bin Faisal University, Dammam, SAU; 5 Dentistry, Umm Al-Qura University, Makkah, SAU; 6 General Dentistry, Majmaah University, Majmaah, SAU; 7 General Dentistry, Cosma Dental Polyclinics, Riyadh, SAU; 8 General Dentistry, Batterjee Medical College, Jeddah, SAU; 9 General Dentistry, Qassim Health Cluster, Ministry of Health, Qassim, SAU; 10 General Dentistry, East Jeddah General Hospital, Ministry of Health, Jeddah, SAU; 11 Orthodontics and Dentofacial Orthopaedics, Al-Noor Specialist Hospital, Makkah, SAU; 12 Orthodontics, Jaber Al-Ahmad Specialist Hospital Dental Centre, Kuwait City, KWT

**Keywords:** airway dimensions, bone-borne rapid palatal expansion, cbct, long-term stability, marpe

## Abstract

This review aimed to synthesize cone-beam CT (CBCT) evidence on changes in upper airway dimensions and postexpansion stability following miniscrew-assisted rapid palatal expansion (MARPE) and other bone-borne expanders. A systematic review was conducted following the Preferred Reporting Items for Systematic reviews and Meta-Analyses 2020 guidelines. PubMed/MEDLINE, Cochrane Library, ScienceDirect, Semantic Scholar, and Google Scholar were searched for English-language human clinical studies published from 2010 to August 2, 2025. Eligible designs included randomized and non-randomized trials and prospective/retrospective cohorts reporting CBCT-derived linear or volumetric nasal and upper airway outcomes with at least five months of follow-up, using pre-post comparisons and/or comparisons with conventional expanders or untreated controls. Two reviewers independently screened studies, extracted data, assessed risk of bias, and evaluated certainty of evidence. Owing to heterogeneity in appliance designs, airway segment definitions, and follow-up schedules, a meta-analysis was not performed, and findings were narratively synthesized. In total, 17 studies (14 unique datasets) involving 12-102 participants per study and ages ranging from 8 to 33 years were included. Most studies demonstrated short- to mid-term increases in nasal cavity width/volume and nasopharyngeal dimensions after MARPE-type expansion, suggesting predominant anterior airway effects. Changes in the oropharyngeal region were inconsistent, with some studies showing enlargement and others showing no measurable difference. Long-term follow-up (≥12 months) generally indicated maintenance of skeletal and airway gains, though partial relapse was reported in some parameters; skeletal relapse tended to be smaller than dental relapse. Overall certainty of evidence for airway outcomes was low to very low. MARPE and related bone-borne expanders may increase nasal and nasopharyngeal airway dimensions and offer relatively stable transverse skeletal widening, but the predictability and clinical durability of airway benefits remain uncertain. Higher quality studies with standardized CBCT protocols and added functional airway assessments are required to confirm long-term clinical relevance.

## Introduction and background

Contemporary orthodontics has evolved toward broader, more functionally oriented practices that recognize the significance of airway health in patients with maxillary deficiency. Modern orthodontic treatment planning now integrates skeletal modifications and considers their impact on the upper airway as a critical component of comprehensive care. Maxillary transverse deficiency, defined by the presence of a narrow upper jaw, often leads to dental crowding, labial flaring of upper incisors, speech difficulties, and, crucially, respiratory limitations. It can be caused by several factors, such as prolonged non-nutritive sucking (e.g., thumb sucking), respiratory obstruction such as enlarged adenoids, atypical swallowing, or a low tongue posture [1]. In simple terms, maxillary transverse deficiency reflects an upper jaw that is too narrow for the dentition and airway, which may compromise both occlusion and nasal breathing.

Rapid maxillary expansion (RME) is a commonly used technique to expand the maxilla in growing patients. It is a tooth-borne appliance that mainly derives anchorage from the posterior teeth. In tooth-borne expanders such as conventional RME, the forces are transmitted primarily through the teeth, and the skeletal response depends on the flexibility of the midpalatal suture and surrounding structures. Separation of the midpalatal suture (MPS) is then facilitated with a Hyrax or Haas-type expander that exerts lateral forces on the palatal bone, which is pliable in growing patients [2]. This widening of the upper jaw helps correct crossbites and, in some cases, enhances nasal airflow. Even so, the success of RME is largely dependent on the stage of maturation of the midpalatal suture. Once the suture has fused, RME is usually contraindicated, as it can lead to expansion failure, buccal tipping of the teeth, root resorption, gingival recession, and relapse [3]. Thus, in skeletally mature patients with non-pliable MPS, surgically assisted rapid palatal expansion (SARPE) is used to achieve maxillary expansion. One traditionally used method is a midpalatal osteotomy between the central incisors, which is then followed by a tooth-borne or a bone-borne expander. Over time, many modifications in osteotomies have been developed based on different theories regarding the location of resistance to expansion. Although it provides positive outcomes, SARPE is not favored by most orthodontists and patients due to its invasive nature, which requires surgery under general anesthesia, leading to increased discomfort, pain, swelling, and higher treatment costs [4].

To overcome these limitations, miniscrew-assisted rapid palatal expansion (MARPE) and other bone-borne expanders were introduced as less invasive alternatives to SARPE in selected late adolescent and adult patients [5,6]. MARPE uses miniscrews or temporary anchorage devices to anchor the expander directly to the palatal bone, so that a larger proportion of the expansion force is expressed skeletally rather than dentally. In contrast to tooth-borne RME, MARPE and related bone-borne designs are therefore considered “skeletal” or hybrid expanders that aim to maximize orthopedic effects and minimize unwanted tooth tipping. Recent studies have found that achieving palatal expansion in late adolescents and adults was also possible, potentially minimizing the need for surgically assisted expansion in suitable patients.

MARPE can also significantly increase the airway volume by simultaneously expanding the nasal cavity, nasopharynx, and alar width. Traditional RME tends to produce a more V-shaped opening, where the anterior palatal region widens more than the posterior [7]. A V-shaped opening indicates greater lateral separation at the dental level than at the nasal floor, whereas a more parallel opening suggests similar transverse widening at both the nasal and palatal levels. This reduces the amount of airway enhancement, especially in the posterior region, as the resistance to airway persists due to inadequate expansion. Conversely, MARPE has shown a more parallel or pyramidal suture opening, even in late adolescents and adults, allowing a more uniform transverse skeletal expansion to be obtained, especially in the posterior nasal region. For clarity, in this context, the “nasal cavity” refers to the space extending from the anterior nasal aperture to the choanae, the “nasopharynx” extends from the choanae to the level of the soft palate, and the “oropharynx” from the soft palate to approximately the level of the epiglottis; these airway segments are commonly analyzed in cone-beam CT (CBCT)-based volumetric studies. These patterns of expansion are associated with increases in nasal and nasopharyngeal dimensions on CBCT; however, such structural changes do not necessarily indicate functional improvement, and their impact on airway resistance remains uncertain.

While few studies have investigated the correlation between airway volume and MARPE and how it significantly increases the airway volume, there is very little evidence on the postexpansion stability of the achieved corrections [8,9]. Existing reports are heterogeneous in terms of appliance design, activation protocols, and follow-up durations, which makes it difficult to determine whether any observed airway improvements are stable skeletal changes or short-term adaptations. Even though certain findings suggest that the improvement can persist, the question arises whether these changes are actually stable or simply a transient adaptation [10,11]. For this reason, the present review focused on studies with a minimum follow-up period of five months, to allow at least a medium-term assessment of postexpansion stability.

As opposed to traditional two-dimensional cephalometric imaging, CBCT provides a more comprehensive three-dimensional view of the craniofacial region. This allows detailed visualization of the midpalatal suture and volumetric assessment of airway structures. Therefore, CBCT-derived measurements offer three-dimensional information on nasal cavity and upper airway morphology that cannot be obtained from conventional two-dimensional radiographs. By reducing anatomical overlap, CBCT offers better clarity for evaluating skeletal airway dimension changes over time. However, its accuracy is influenced by factors such as radiation dose considerations, segmentation methods, and threshold variability. Despite these limitations, CBCT remains the most widely used modality in current MARPE research for assessing both short- and long-term changes, which is why this review includes studies that rely on CBCT for evaluation.

At present, there is a gap in evidence regarding the durability of airway improvements post-MARPE. Based on this, the research question for this systematic review is as follows: What is the impact of bone-borne and MARPE on postexpansion airway dimensions and stability on CBCT, as assessed in pre-post studies and in comparison with conventional expansion or controls? The underlying hypothesis of this review is that bone-borne and MARPE produce significant increases in nasal and nasopharyngeal dimensions that remain at least partially stable over time and are greater than those achieved with conventional tooth-borne expansion or no expansion.

## Review

Methodology

This systematic review was conducted in accordance with the Preferred Reporting Items for Systematic Reviews and Meta-Analyses (PRISMA) 2020 guidelines to ensure consistency and reproducibility [12].

Eligibility Criteria

According to the Population, Interventions, Comparisons, Outcome, Study design (PICOS) strategy, studies were included if they met the following criteria.

Population (P): Developing and skeletally mature individuals of both sexes who had undergone MARPE. The general population and individuals treated with conventional RME were also considered for comparative analysis.

Intervention (I): MARPE or any bone-borne variants such as maxillary skeletal expanders (MSEs).

Comparison (C): Baseline versus post-treatment comparisons (pre- and post-MARPE) were included. Comparative studies involving conventional RME were also considered.

Outcome (O): Linear and volumetric CBCT-derived measurements of nasal cavity and upper airway dimensions, with a minimum follow-up period of five months. For consistency in reporting, follow-up durations were categorized as short-term (≤6 months), mid-term (6-12 months), and long-term (≥12 months).

Study Design (S): Eligible studies included randomized and non-randomized clinical trials, cohort studies, retrospective studies, and case-control studies published from 2010 to 2025. A detailed summary of the PICO (Population, Intervention, Comparison, Outcome) criteria used in this review is provided in Table 1.

**Table 1:** PICOS framework for the systematic review. PICOS = Population, Interventions, Comparisons, Outcome, Study design; MARPE = miniscrew-assisted rapid palatal expansion; MSE = maxillary skeletal expander; RME = rapid maxillary expansion; OPG = orthopantomogram

Inclusion criteria	Exclusion criteria
Population (P)	
Developing and skeletally mature individuals treated with MARPE; males and females	Patients with cleft lip, craniofacial anomalies, or syndromic conditions
Intervention	
MARPE, MSE, or any bone-borne variants	Adjunctive procedures with MARPE, such as maxillary protraction with face masks
Comparison	
RME or no intervention; pre- and post-MARPE evaluations	Surgically assisted rapid palatal expansion (SARPE/SARME)
Outcome	
CBCT analysis of airway dimensions, ≥5 months follow-up period.	Panoramic radiographs (OPGs), lateral cephalograms, acoustic rhinometry, or rhinomanometry-based airway resistance assessments
Study design	
Randomized and non-randomized clinical trials, cohort, retrospective, and case-control studies published between 2010 and 2025	In vitro, animal studies, finite element analyses, narrative reviews, case reports, and expert opinions

To ensure the validity and reliability of this systematic review, only peer-reviewed articles published in English were included. Eligible studies comprised original clinical research, including randomized controlled trials (RCTs), prospective and retrospective cohort studies, case-control studies, and case series that evaluated the effects of MARPE on upper airway dimensions using CBCT.

In vitro studies, animal studies, finite element analyses, narrative reviews, case reports, and expert opinions were excluded. Studies involving patients with craniofacial anomalies, cleft palate, or syndromic conditions were also excluded. Additionally, investigations combining MARPE protocols with adjunctive interventions, such as surgically assisted expansion or maxillary protraction using face masks, were not included. These exclusions were made to maintain methodological uniformity and focus the analysis on the isolated effects of MARPE in human clinical studies.

Information Sources and Search Strategy

Two independent reviewers performed a comprehensive literature search in PubMed/MEDLINE, Cochrane Library, ScienceDirect, and Semantic Scholar, along with Google Scholar for grey literature. The search covered studies published from January 2010 to August 2, 2025, with the final search update completed on August 2, 2025. This time frame (January 2010 to August 2025) was chosen because CBCT-based airway assessment and miniscrew-assisted maxillary expansion began to be consistently reported in the literature during this period. In addition, the reference lists of all included articles and key orthodontic and CBCT-focused journals were hand-searched to identify any additional eligible studies that might not have been retrieved through the electronic search.

The preliminary keywords used were “MARPE,” “airway volume,” “CBCT,” “nasopharynx,” and “orthodontics.” MeSH terms such as “Palatal Expansion Technique,” “Airway Resistance,” and “Cone-Beam Computed Tomography” were incorporated using Boolean operators (AND, OR) to refine the search. Filters were applied to limit results to human studies, articles published from 2010 onwards, and those published in English. The main search terms and Boolean combinations are summarized in Table 2.

**Table 2:** Detailed search strategy across bibliographic databases and grey literature sources.

Database	Search interface	Search string	Filters applied	Number of hits
PubMed	Advanced search builder	“Palatal expansion Technique” [MeSH] OR “maxillary expansion” OR “MARPE” OR “miniscrew assisted rapid palatal expansion”) AND (“airway” OR “nasopharynx” OR “nasal cavity” OR “upper airway”) AND (“Cone-Beam Computed Tomography” [MeSH] OR “CBCT”	English language, human studies, 2010–2025	786
Cochrane Library	Search manager	(“palatal expansion technique” OR “maxillary expansion” OR “MARPE” OR “miniscrew assisted rapid palatal expansion”) AND (“airway” OR “upper airway” OR “nasopharynx” OR “nasal cavity”) AND (“volume” OR “volumetric” OR “cone-beam computed tomography” OR “CBCT”)	English language, human studies, 2010–2025	455
Semantic Scholar	Basic keyword search	“miniscrew assisted rapid palatal expansion” OR “MARPE” OR “maxillary skeletal expander” OR “bone-borne rapid maxillary expansion” AND (“airway” OR “nasal cavity” OR “nasopharynx” OR “upper airway”) AND (“CBCT” OR “cone beam computed tomography”	English language, human studies, 2010–2025	185
ScienceDirect	Basic keyword search	“miniscrew assisted rapid palatal expansion” OR “MARPE” OR “maxillary skeletal expander” OR “bone-borne rapid maxillary expansion” AND (“airway” OR “nasopharynx” OR “nasal cavity” OR “upper airway”) AND (“CBCT” OR “cone beam computed tomography”)	English language, human studies, 2010–2025	328
Google Scholar	Advanced search	“miniscrew assisted rapid palatal expansion” OR “MARPE” AND “airway” AND “CBCT”	English language, 2010–2025	661

Selection Process

To minimize the risk of individual bias, two independent reviewers screened all titles and abstracts to identify studies that met the eligibility criteria. Data extraction was then conducted independently by both reviewers. Based on the predefined inclusion criteria, relevant data were extracted from the full-text articles, including authors, year of publication, study design, age, sex, sample size, type of appliance used, treatment duration, follow-up period, measurement methods, and outcomes. Final inclusion was determined after thorough reading and re-evaluation by both reviewers. Any discrepancies were resolved through discussion until a consensus was reached.

Data Extraction

Data extraction was performed manually using a predefined table. Only numerical values explicitly reported in the studies (mean, standard deviation, and sample size) were collected. When SDs or numerical values were missing, the study was included only in the qualitative synthesis. All extracted data were checked twice for accuracy and unit consistency.

Risk of Bias Assessment

The risk of bias for the included studies was assessed according to their respective study designs. For RCTs, the Cochrane Risk of Bias tool version 2 (RoB 2) [13] was employed. It evaluated the following five domains: (1) bias arising from the randomization process, (2) deviations from intended interventions, (3) missing outcome data, (4) measurement of the outcome, and (5) selection of the reported result.

For non-randomized studies, including both prospective and retrospective observational designs and secondary analyses of randomized datasets, the Risk of Bias in Non-randomized Studies of Interventions (ROBINS-I) tool was utilized [14]. This tool assesses bias across the following seven domains: confounding, participant selection, classification of interventions, deviations from intended interventions, missing data, measurement of outcomes, and selection of the reported result.

For studies based on secondary analysis of RCT data, the ROBINS-I tool was used. In addition, particular attention was paid to potential biases introduced by selective outcome reporting or incomplete access to the original dataset.

Each included study was independently assessed by two reviewers. Any disagreements were resolved through discussion, and if consensus was not achieved, a third reviewer was consulted. The overall risk of bias judgments was presented as “low risk,” “some concerns,” or “high risk” for each domain and summarized in tabular and graphical formats using the ROBVIS tool [15].

Effect Measures

Due to substantial heterogeneity in measurement units, CBCT protocols, appliance designs, and follow-up durations, a narrative synthesis was conducted. Although continuous outcomes such as airway volume and linear dimensions were extracted, no pooled mean difference/standardized mean difference estimates were calculated, and no forest plots were generated.

Synthesis Methods

Data extracted from the included studies were first tabulated to summarize study characteristics, participant demographics, type of appliance used, expansion protocol, follow-up duration, and key outcomes.

A meta-analysis was not feasible due to several reasons, including high clinical and methodological heterogeneity among the included studies in terms of study design, sample characteristics, and intervention protocols. There were also variations in CBCT measurement parameters, with differences in airway segmentation techniques, threshold values, and software used. Inconsistent outcome reporting was another issue, as some studies presented outcomes in different units (mm³, cm³, or percentage change), limiting direct comparability. Additionally, there was a limited number of studies with comparable outcome measures across similar time frames. Given these factors, statistical pooling of the data would have produced misleading summary estimates. Therefore, the results were synthesized narratively, focusing on the direction of effect, magnitude, and overall trends in airway stability following MARPE.

Reporting Bias Assessment

Reporting bias was assessed at both the study level and the review level. For RCTs, selective reporting bias was evaluated using the corresponding domain in the RoB 2 tool, while for non-randomized studies, it was assessed using the ROBINS-I domain on “selection of the reported result.”

Certainty Assessment

The certainty of evidence for each outcome was evaluated using the Grading of Recommendations, Assessment, Development, and Evaluation (GRADE) approach [16]. In this approach, the certainty level was downgraded or upgraded based on five domains, namely, risk of bias, inconsistency, indirectness, imprecision, and publication bias. Two reviewers independently assessed these domains for each outcome, and discrepancies were resolved by consensus. The overall certainty of evidence was categorized as high, moderate, low, or very low, according to the GRADE framework.

Results

Study Selection

**Figure 1:**
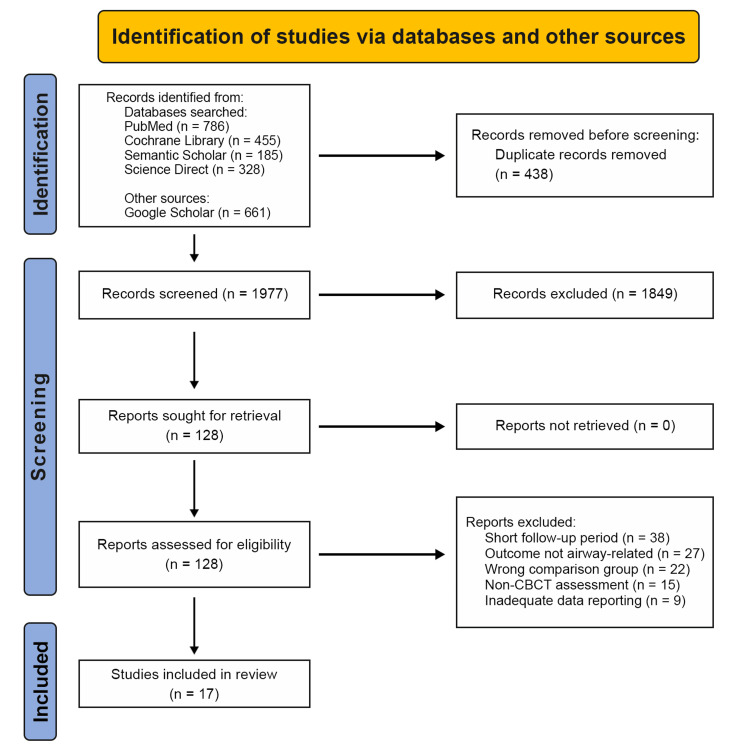
Preferred Reporting Items for Systematic Reviews and Meta-Analyses (PRISMA) flow diagram.

In total, 2,415 records were identified through database searches. After removing 438 duplicate records, 1,977 unique records were screened based on titles and abstracts. Of these, 1,849 records were excluded for not meeting the inclusion criteria. The remaining 128 full-text reports were assessed for eligibility. Of these, 111 reports were excluded due to short follow-up periods, outcomes not related to the airway, wrong comparison groups, non-CBCT assessments, and inadequate data reporting. Finally, 17 studies met the inclusion criteria and were included in the systematic review. The study selection process is illustrated in Figure 1.

Study Characteristics

A total of 17 publications [17-33], representing 14 unique datasets, were included in this review. Among these, four were primary RCT datasets. Additionally, four publications were identified as retrospective secondary analyses of RCT datasets: three [21,22,23] originated from the same parent RCT, and one [24] was derived from a separate randomized trial. The other nine studies consisted of six retrospective cohort studies and three prospective cohort studies. The sample sizes ranged from 12 to 102 participants, with ages spanning from 8 to 33 years, thereby including both adolescent and adult populations.

Among the included studies, six evaluated pre- and postexpansion changes following MARPE [25,26,29,31-33]. Eight studies compared MARPE with conventional expanders or untreated control groups [18-25]. One study compared two types of micro-osteoperforation methods (MOPs) and their effects on expansion, while another assessed the influence of different activation protocols (slow vs. rapid) in MARPE [17,30].

The minimum follow-up period was five months. Among the included studies, three evaluated short-term outcomes (≤6 months) [28-30], seven reported mid-term outcomes (6-12 months) [17,18,20,24,25,27,32], and seven provided long-term follow-up data (≥12 months) [19,21-23,26,31,33]. A summary of the included studies is shown in Table 3.

**Table 3:** Summary of included studies. Secondary analyses from one parent RCT dataset (Mehta et al., 2022; Empson et al., 2023; Ahmida et al., 2023) were counted once for synthesis. The Garib et al. (2021) study represents an independent secondary analysis of a different parent RCT dataset and was also counted only once. RCT = randomized controlled trial; MARPE = miniscrew-assisted rapid palatal expansion; RPE = rapid palatal expansion

Author (year)	Study design	Sample size	Age (mean ± SD/range)
Alawady et al. (2025) [17]	RCT	18	Group I: 19.57 ± 1.81 years; Group II: 19.13 ± 1.25 years
Shendy et al. (2022) [18]	RCT	30	11–17 years
Bazargani et al. (2020) [19]	RCT	54	8–13 years
Celenk-Koca et al. (2018) [20]	RCT	40	Group 1: 13.84 ± 1.36 years; Group 2: 13.81 ± 1.23 years
Mehta et al. (2022) [21]	Retrospective secondary analysis of RCT dataset*	60	11–15 years
Empson et al. (2023) [22]	Retrospective secondary analysis of RCT dataset*	60	11–15 years
Ahmida et al. (2023) [23]	Retrospective secondary analysis of RCT dataset*	60	MARPE: 13.7 ± 1.74 years; RPE: 13.9 ± 1.14 years; control: 13.3 ± 1.49 years
Garib et al. (2021) [24]	Retrospective secondary analysis of RCT dataset*	40	9–13 years
Lee et al. (2022) [25]	Retrospective study	28	20.4 ± 7.3 years
Kim et al. (2018) [26]	Retrospective study	14	18.3–26.5 years (22.7 ± 3.3 years)
Oh et al. (2019) [27]	Retrospective study	102	≤18 years
Yacout et al. (2022) [28]	Retrospective study	20	12–16 years
Sim et al. (2023) [29]	Retrospective study	17	13.29 years (22.3 ± 7.9 years)
Yacout et al. (2021) [30]	Prospective study	24	12–16 years
Tang et al. (2021) [31]	Retrospective study	31	18–33 years (22.14 ± 4.76 years)
Al-Mansour et al. (2022) [32]	Prospective study	12	18–30 years
Chen et al. (2025) [33]	Prospective study	31	≥16 years (mean age = 26.2 years)

Risk of Bias in Studies

Risk of bias in studies was assessed based on the study design. Four primary RCT datasets were assessed using the RoB 2.0 tool. Four additional articles [21-24] represented secondary analyses of previously randomized cohorts, without new random allocation or prospective conduct, and were therefore evaluated as non-randomized using the ROBINS-I tool. The remaining non-randomized studies (six retrospective and three prospective cohorts) were also assessed using the ROBINS-I tool, as they lacked randomization and were more vulnerable to confounding.

Most studies utilized CBCT for outcome evaluation, allowing relatively objective and standardized measurement. Intra- and inter-examiner reliability was frequently reported through repeated assessments on randomly selected data. However, blinding of assessors was rarely feasible due to the obvious radiographic presence of skeletal expansion, contributing to a moderate risk of detection bias across several studies.

Studies that employed a pre vs. post design and studies without a control group were judged to have a serious risk of confounding, as growth-related anatomical changes could not be separated from the effects of MARPE [25-33]. The absence of comparator groups also prevented proper analysis of the effects. Bias due to the selection of participants was frequently identified among the non-randomized studies. Furthermore, many retrospective cohorts included only patients who completed MARPE and had complete radiographic records, without documentation of drop-outs or treatment failures [25-32]. This selective inclusion could lead to overestimation of airway improvements, as the final sample may represent individuals who responded favorably to treatment. Lack of transparency regarding excluded cases and baseline differences between included and non-included patients led to a judgment of moderate to serious risk of bias in this domain.

Bias in the classification of interventions was generally low because the device type and the timing of expansion were well documented in most studies. Variations in activation protocols, particularly those tailored to different age groups, may have influenced expansion and subsequent airway improvement. Because such deviations were related to baseline patient characteristics and not analytically adjusted for, studies implementing age-dependent activation protocols were assigned a moderate risk of bias due to deviations from intended interventions [25]. Selective inclusion of only patients completing treatment with both pre- and post-CBCT scans, without clear accounting for excluded or unsuccessful cases, introduced a serious risk of bias due to missing data in some retrospective cohorts [27]. The missingness is likely related to treatment response. Additionally, several non-randomized studies lacked transparent statistical control for key confounders such as skeletal maturity, baseline airway volume, or respiratory conditions, further elevating the overall risk of bias.

**Figure 2:**
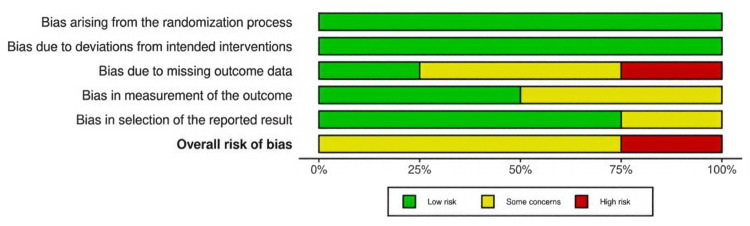
Summary of risk of bias across randomized trials. A domain-level summary plot illustrating the proportion of studies rated as low risk (green), some concerns (yellow), or high risk (red) across the five RoB 2.0 domains, i.e., bias arising from the randomization process, bias due to deviations from intended interventions, bias due to missing outcome data, bias in measurement of the outcome, and bias in selection of the reported result. The overall risk of bias for each domain is displayed in the final row. RoB 2.0 = Risk of Bias version 2

Overall, the risk of bias was judged to be serious in the majority of included studies, particularly among the retrospective cohorts. Key contributing factors included confounding, selective participant inclusion, and lack of blinding in outcome assessment. Although the evidence was suitable for qualitative synthesis, the conclusions should be interpreted with caution due to the inherent limitations of the available study designs. The distribution of domain-specific and overall risk of bias for the randomized trials is summarized in Figure 2.

**Figure 3:**
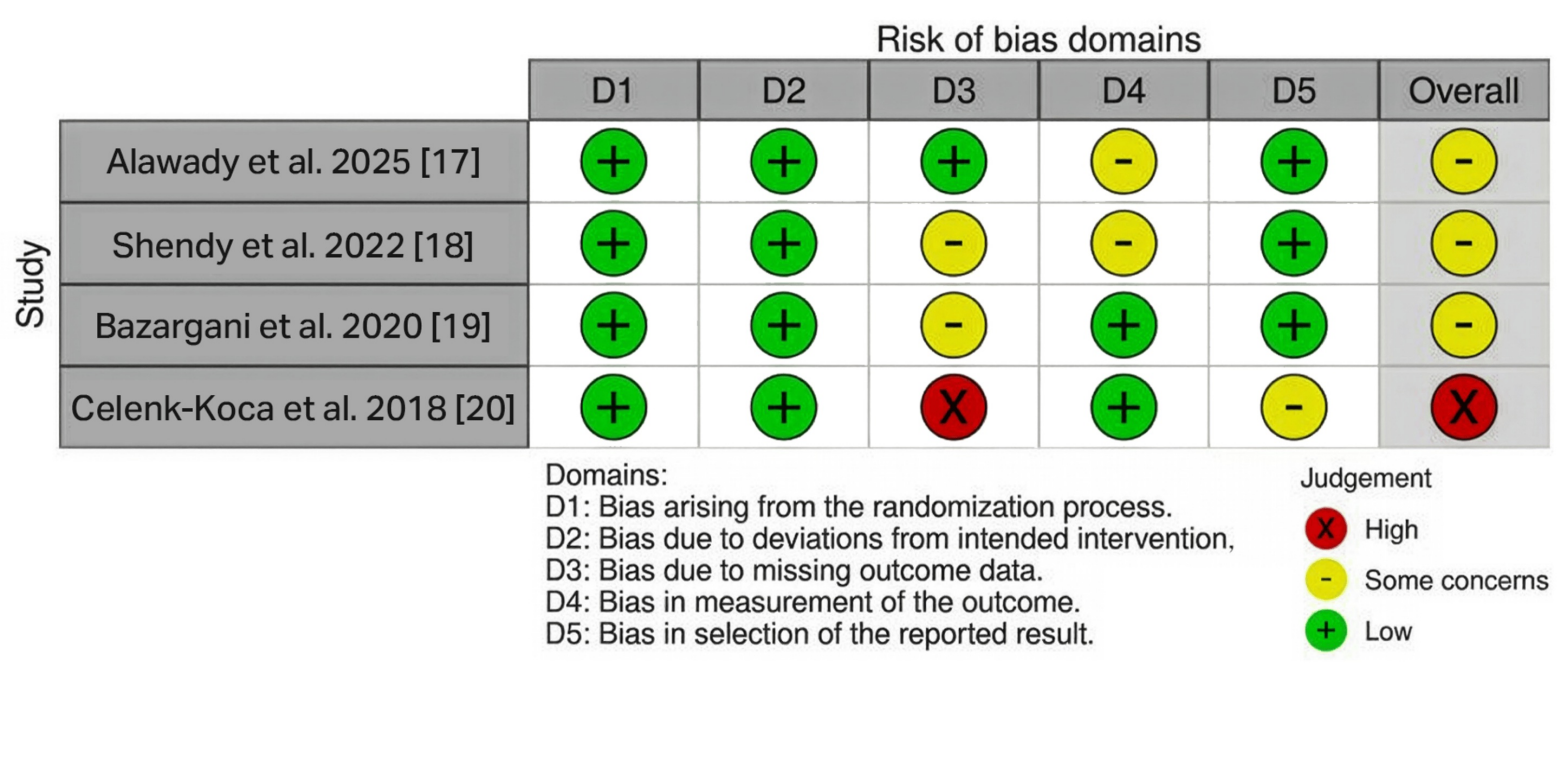
Study-level risk of bias assessment for randomized trials. Traffic-light plot showing the RoB 2.0 judgments for each included randomized trial across the five domains: D1: bias arising from the randomization process; D2: bias due to deviations from intended interventions; D3: bias due to missing outcome data; D4: bias in measurement of the outcome; D5: bias in selection of the reported result. Green = low risk; yellow = some concerns; red = high risk. The rightmost column represents the overall risk of bias judgment for each study. RoB 2.0 = Risk of Bias version 2

The study-level traffic-light plot for the included RCTs, assessed using the Cochrane RoB 2 tool, is presented in Figure 3.

**Figure 4:**
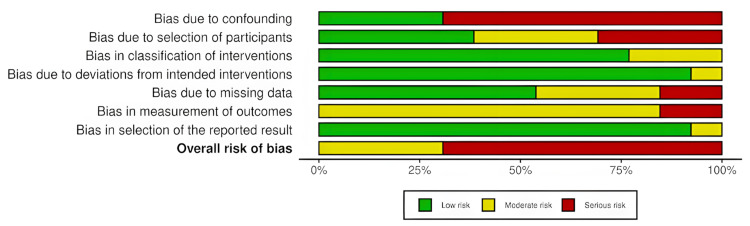
Summary of risk of bias in non-randomized studies assessed with ROBINS-I. Domain-level summary plot showing the proportion of non-randomized studies (retrospective and prospective cohorts) judged as low risk (green), moderate risk (yellow), or serious risk (red) of bias across ROBINS-I domains, i.e., bias due to confounding, bias due to selection of participants, bias in classification of interventions, bias due to deviations from intended interventions, bias due to missing data, bias in measurement of outcomes, and bias in selection of the reported result. The final row represents the overall risk of bias judgment. ROBINS-I = Risk of Bias in Non-randomized Studies of Interventions

The overall distribution of risk of bias across the non-randomized studies, assessed using the ROBINS-I tool, is presented in Figure 4.

**Figure 5:**
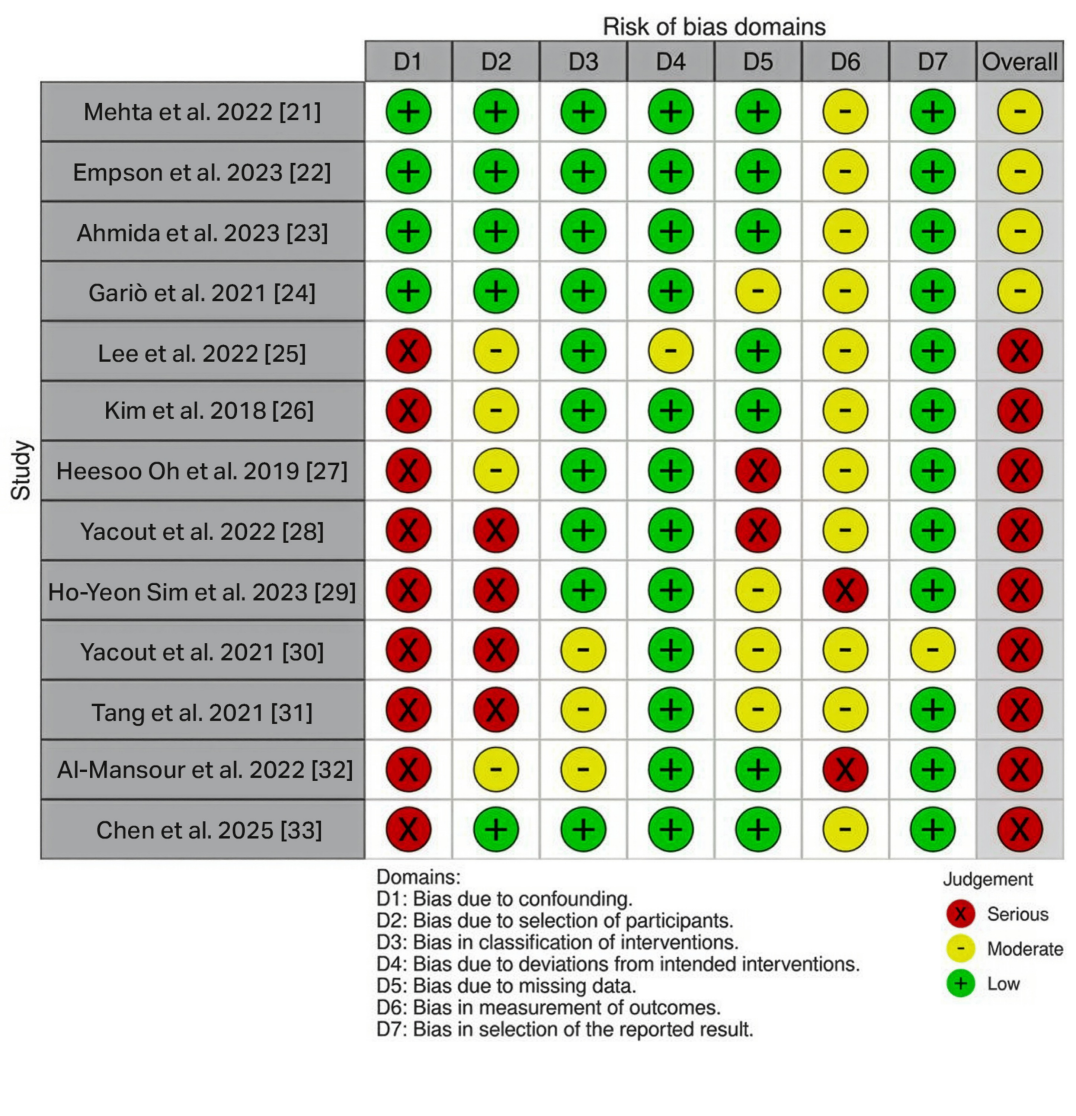
Study-level risk of bias assessment for non-randomized studies using the ROBINS-I tool. Traffic-light plot showing risk-of-bias judgments for each non-randomized study (retrospective and prospective cohorts) across the seven ROBINS-I domains: D1: bias due to confounding; D2: bias due to selection of participants; D3: bias in classification of interventions; D4: bias due to deviations from intended interventions; D5: bias due to missing data; D6: bias in measurement of outcomes; D7: bias in selection of the reported result. Green = low risk; yellow = moderate risk; red = serious risk. The rightmost column displays the overall risk of bias judgement for each study. ROBINS-I = Risk of Bias in Non-randomized Studies of Interventions

The study-level traffic-light plot for the non-randomized studies, assessed using the ROBINS-I tool, is presented in Figure 5.

Results of individual studies

Overall, most studies reported increases in transverse skeletal and airway dimensions following expansion, although the magnitude and statistical significance varied between studies and according to appliance design, activation protocol, and follow-up duration.

Comparison Among Expander Types

Shendy et al. (2022) compared conventional Hyrax, hybrid Hyrax, and MSE appliances and found the highest mean increases in total airway, nasopharynx, retropalatal, and retroglossal volumes in the MSE group (18.76%, 19.75%, 9.41%, and 12.75%, respectively). The greatest percentage increase in nasal cavity volume was again observed with MSE (22.73%), followed by hybrid and conventional expanders (both 7.86%) [18]. Similarly, Bazargani et al. (2020) reported a mean nasal cavity width increase of 3.5 mm in the tooth-bone-borne (TBB) expander group, which was nearly twice that of the tooth-borne group (1.8 mm). The stability of expansion one year post-treatment was comparable between both groups. They concluded that while tooth-borne RME may be sufficient for younger patients without airway obstruction, TBB expanders offered superior skeletal effects in those presenting with airway compromise [19].

Celenk-Koca et al. (2018) also found significantly greater nasal cavity width expansion in the molar region (NCWM = 2.9 ± 1.7 mm) with bone-borne expanders compared to conventional appliances (NCWM = 1.2 ± 1.1 mm), although premolar changes were not statistically significant [20]. Garib et al. (2021) similarly reported greater nasal cavity width in the hybrid-supported expander group (28.81 ± 2.35 mm) compared with conventional Hyrax (28.33 ± 2.18 mm) [24].

Oh et al. (2019) found that the MSE group showed significantly greater nasal cavity width changes (w_LN = 1.38 ± 1.6 mm, w_IN = 2.66 ± 1.25 mm) compared with the bone-anchored maxillary expander (w_LN = 0.91 ± 1.1 mm; w_IN = 1.22 ± 1.7 mm) and tooth-anchored maxillary expander (w_LN = 1.16 ± 1.05 mm; w_IN = 1.26 ± 1.82 mm) groups (p = 0.002). In addition, they discovered greater skeletal displacement at the nasal floor, maxillary base, and midpalatal suture in the MSE group, with a near-parallel suture opening pattern [27].

Influence of Activation Protocol

Yacout et al. (2022) compared slow (SME) and rapid (RME) activation protocols of miniscrew-assisted expansion and found that both groups showed significant increases in anterior, middle, and posterior nasal cavity width. However, SME demonstrated a significantly greater increase in anterior nasal cavity width compared to RME (mean difference = 2.64 mm; 95% confidence interval = 0.83-4.45; p = 0.007) [28].

In a related study, Yacout et al. (2021) reported that slow activation of MARPE resulted in a significant increase in nasal cavity width at the maxillary first premolar (2.95 mm) and first molar (2.62 mm) levels (p < 0.001). Rapid activation also showed significant increases of 2.54 mm (first premolar) and 2.57 mm (first molar) (p < 0.001) with no significant difference between protocols. In the slow expansion group, 46.6% of jackscrew expansion produced skeletal nasal widening. This figure was 43.6% in the rapid group, suggesting comparable skeletal efficiency [30].

Micro-Osteoperforations and Expansion Patterns

Alawady et al. (2025) compared the use of MSE with midpalatal MOPs only and MSE with both midpalatal and buccal MOPs in young adults. They found no significant differences in lower nasal section and upper nasal section measurements between groups; however, both groups demonstrated statistically significant pre- and postexpansion increases [17]. The mean midpalatal suture split was 3.96 mm at the anterior nasal spine (ANS) and 3.04 mm at the posterior nasal spine (PNS) in the midpalatal-only MOP group, while the midpalatal buccal group achieved 3.8 mm (ANS) and 3.17 mm (PNS). This reflected an almost parallel suture opening (PNS/ANS ≈ 83%) with a trapezoidal expansion pattern and a greater anterior than posterior opening.

The authors concluded that both techniques effectively produced skeletal widening consistent with prior finite element models, but adding buccal MOPs did not significantly enhance expansion. This suggests stress reduction and displacement facilitation near the suture with adjunctive MOPs.

Long-Term Stability and Skeletal Correlations

Tang et al. (2021) observed a significant positive correlation between PCBT and postexpansion change (T2-T1) in nasal floor width (NF), implying that patients with thicker cortical bone exhibited less relapse. Furthermore, significant increases were seen in NF (2.33 mm) and N-N (2.26 mm) between T0 and T1. The smallest width decrease occurred at the nasal cavity level and measured only 0.13 mm, which represented 5.75% of total expansion, indicating high stability. The authors also noted that these changes could be accompanied by alar base widening, potentially influencing nasal aesthetics [31].

Chen et al. (2025) reported a mean immediate skeletal maxillary expansion (ΔNFW T0-T1) of 3.7 ± 1.5 mm, with a minor relapse of 0.6 ± 1.2 mm (11.6%) during follow-up. The mean nasal cavity width increased from 2.1 ± 1.4 mm immediately after expansion to 2.3 ± 1.6 mm at follow-up (p = 0.34), confirming stable nasal widening with minimal regression [33].

Airway Volumetric and Nasal Septum Changes

Data from a prior randomized trial were analyzed by three studies: Mehta et al. (2022), Empson et al. (2023), and Ahmida et al. (2023). Mehta et al. (2022) found a significant reduction in nasal septal deviation angle (NSDA, p = 0.007) and greater posterior nasal cavity width increase in MARPE (1.75 mm) than in RPE (0.78 mm) and controls (0.63 mm) [21-23]. Empson et al. (2023) noted significant short-term changes in facial landmarks such as pogonion, gonion, and alar base in MARPE and RPE groups relative to controls, although these differences equalized over the long term [22]. Ahmida et al. (2023) observed significant short-term increases in right and left nasomaxillary sutures (p = 0.048 and 0.029), but no long-term intergroup differences, except for greater midpalatal suture widening at incisor, canine, and molar levels in MARPE-treated subjects [23].

Lee et al. (2022) reported significant alleviation of nasal septal deviation (NSD) and increases in nasal septal length and lateral nasal wall width; all changes were maintained at the six-month follow-up [25]. Kim et al. (2018) also documented a nasal cavity volume increase from 10,822.6 mm³ to 12,532.8 mm³ immediately postexpansion (Δ = 1,061.6 mm³, p < 0.05) and a further gain of 648.6 mm³ at one year (total Δ = 1,710.2 mm³, p < 0.05). The total airway volume increased by 2,652.6 mm³ (p < 0.05), with significant cross-sectional area gains in anterior and middle airway segments (14.6 mm² and 31.6 mm², respectively; p < 0.05) [26].

Retention Period and Airway Stability

Sim et al. (2023) observed a significant increase in inferior nasal meatus volume between T0-T1 (436.01 ± 352.54 mm³; p < 0.001) and T0-T2 (443.58 ± 293.36 mm³; p < 0.001), with no significant difference between T1-T2, indicating stable retention of airway enlargement. The tortuosity ratio (TR) showed no significant change across all time intervals, suggesting negligible morphological alterations in the nasal septum. The linear measurement between the nasal septum (C point) and the lateral nasal wall increased significantly after MARPE, with percentage gains at T1 of 7.34 ± 3.91 and 7.73 ± 4.35 at T2 in the concave group, and 6.05 ± 3.62 at T1 and 6.32 ± 3.54 at T2 in the convex group [29].

Al-Mansour et al. (2022) also reported significant volumetric gains: mean nasal volume increased from 20.81 ± 3.23 mm³ (T0) to 23.34 ± 2.97 mm³ (T1) and 23.62 ± 2.85 mm³ (T2), with mean changes of 2.53 ± 1.53 (T0-T1), 0.28 ± 1.10 (T1-T2), and 2.81 ± 1.66 (T0-T2). All changes were statistically significant (p < 0.001). Additionally, pharyngeal volume increased by 2.08 ± 1.73 mm³ (T0-T2; p = 0.014), and total airway volume increased by 4.89 ± 1.76 mm³ (T0-T2; p = 0.014), confirming a consistent long-term airway improvement [32]. In another study, Yacout et al. reported that retroglossal and retropalatal airway dimensions remained unchanged between T1 and T2 in both slow and rapid expansion groups, indicating stability after initial postexpansion remodeling [28]. The characteristics and main findings of each included study are summarized in Table 4.

**Table 4:** Summary of interventions, follow-up duration, retention protocols, airway parameters assessed, and main outcomes for the included studies. Follow-up categories: short-term = ≤6 months; mid-term = 6–12 months; long-term = ≥12 months. Time points: T0/T1 = baseline or pre-expansion; T2 = post-expansion; T3 = follow-up, as reported in each study. MSE = maxillary skeletal expander; MOPs = micro-osteoperforations; MPS = midpalatal suture; MPB = midpalatal suture and buccal sides; RME = rapid maxillary expansion; TB = tooth-borne; TBB = tooth–bone-borne; MARPE = miniscrew-assisted rapid palatal expansion; RPE = rapid palatal expansion; CBCT = cone-beam CT; NCWP/NCWM = nasal cavity width at premolar/molar region; NS = nasal septum; NF = nasal floor; NCW-4, NCW-6 = nasal cavity width at premolar and molar levels; INM = inferior nasal meatus; TR = tortuosity ratio; TAME = traditional tooth-anchored maxillary expander; BAME = bone-anchored maxillary expander

Author (year)	Study groups (intervention vs. comparison)	Follow-up duration				Retention protocol	Airway parameters assessed	Outcome
			Short-term (≤6 months)	Mid-term (6–12 months)	Long-term (≥12 months)			
Alawady et al. (2025) [17]	Group I = MSE with MOPs at midpalatal suture only (MPS); Group II = MSE with MOPs in both MPS and buccal sides (MPB)	Before expansion (T1)	-	After 6 months of RME (T2)	-	6 months: screw retained using flowable composite and stabilized using a ligature wire	Linear transverse measurements in the lower and upper nasal sections	Significant increase in nasal width in the lower and upper nasal sections, no significant intergroup difference
Shendy et al. (2022) [18]	Group 1 = conventional Hyrax; group; 2 = hybrid Hyrax; group; 3 = MSE	Before expansion (T1)	-	After 6 months of RME (T2)	-	6 months: screw stabilized with ligature wire and masked with flowable composite	Total volume, nasopharynx volume, retropalatal volume, retroglossal volume, nasal cavity volume	Significant increase in total, nasopharyngeal, retropalatal, and nasal cavity volumes (p < 0.05) in all groups. ↑Retroglossal volume in conventional and hybrid Hyrax, but not in MSE (p = 0.060). Intergroup comparison revealed no significant differences except for retroglossal volume, which was highest in MSE (p = 0.002)
Bazargani et al. (2020) [19]	TB vs. TBB RME	Before expansion (T0)	After expansion (T1)	-	1 year after expansion (T2)	6 months (method not specified)	N1 = nasal width at the most inferior part of cavum nasi; N2 = nasal width at the widest part of cavum nasi	↑ N1 and N2 in the TBB group compared to TB at T1 and T2, indicating greater nasal width and airway gain (p < 0.05)
Celenk-Koca et al. (2018) [20]	Conventional vs. miniscrew- supported maxillary expansion appliance	Before expansion (T1)	-	6 months following passive retention (T2)	-	6 months passive retention using the same appliance	Nasal cavity width measurement performed on the nasal floor between the maxillary first premolars (NCWP) and maxillary first molars (NCWM)	↑ NCWP and NCWM in conventional and bone-borne groups. Significant increase in NCWM in the bone-borne group compared to the conventional group
Mehta et al. (2022) [21]	Group 1 = MARPE; Group 2 = RPE; Group 3 = controls (no expansion)	Pretreatment (T1)	Postexpansion (T2)	-	Post-treatment (MARPE - 2 years 8 months; RPE - 2 years 9 months; Control - 2 years 7 months) (T3)	Not mentioned	Nasal height (NH), nasal length (NL), nasion -ANS height (NAH), alar width (AW), alar base width (ABW), nasal septal deviation angle (NSDA), posterior and anterior nasal cavity with (PNCW and ANCW)	Significant increase in ABW, NSDA, PNCW, ANCW at T2 and NH, NL, NAH, APL, NSDA, PNCW, ANCW at T3 in the MARPE group. Significantly greater increase in NSDA, PNCW, ANCW in the MARPE compared to the control group
Empson et al. (2023) [22]	Group 1 = MARPE; Group 2 = RPE; Group 3 = controls (no expansion).	Pretreatment (T1)	Postexpansion (T2)	-	Post-treatment (MARPE - 2 years 8 months; RPE - 2 years 9 months; Control - 2 years 7 months) (T3)	Not mentioned	Transverse alar width: distance between the right and left alar bases with anterior cranial base as reference	Significantly increased linear measurements of right alar base in x and y axes and left alar base in x axis at T3 and T2 MARPE group. Significantly greater increase in right and left alar base in the MARPE group compared to control, NS compared to RPE
Ahmida et al. (2023) [23]	Group 1 = MARPE; Group 2 = RPE; Group 3 = controls (no expansion)	Pretreatment (T1)	Postexpansion (T2)	-	Post treatment (MARPE - 2 years 8 months; RPE - 2 years 9 months; Control - 2 years 7 months) (T3)	Not mentioned	Nasomaxillary suture, frontomaxillary suture, zygomaticomaxillary suture, pterygomaxillary suture, midpalatal suture	Significant increase at T2 in frontonasal suture, frontomaxillary suture, nasomaxillary suture, midpalatal suture, both right and left; at T3 in MPS. No significant difference in three groups
Garib et al. (2021) [24]	Hybrid miniscrew-supported expander (HH) vs. conventional Hyrax (CH) expander	Before expansion (T1)	-	11 months postexpansion (T2)	-	11 months retention by maintaining the expander in the oral cavity	Nasal cavity width	Significantly greater increase in nasal cavity widths in HH group compared to CH group. (p = 0.004)
Lee et al. (2022) [25]	Maxillary skeletal expander (pre and post-CBCT comparison)	Pre-expansion (T1)	Post-expansion (T2)	6 month after MSE (T3)	-	Not mentioned	Nasal septal angles (US, MS_C and LS) in the coronal plane; nasal septal angles (AS, MS_A, and PS) in the axial plane; lateral nasal wall angles and widths, and nasal floor (NF) angle; nasal septal length (NSL0 in the coronal plane (between US and LS); NSL in the axial plane (between AS and PS)	Significantly increased vertical and horizontal NS length. NF canting in the opposite direction to NSD. Changes in MS_C and LS angles improved NSD more than changes in the US angle
Kim et al. (2018) [26]	Miniscrew-assisted rapid maxillary expansion (MARME) (pre- and post-CBCT)	Before expansion (T1)	Postexpansion (T2)	-	1 year after expansion (T3)	15.1 weeks: MARME appliance retained	Volume - nasal cavity volume, nasopharynx volume, total airway volume. Area - ANS-perp plane, choanae plane, C3 plane	Significantly increased nasal cavity volume, nasopharyngeal volume and total volume at T2 and additional increase at T3
Oh et al. (2019) [27]	TAME vs. BAME (Dresden-type Hyrax expander)	Before expansion (T1)	-	6 months postexpansion (T2)	-	6 months passive retention using same appliance	Nasal floor, maxillary base, palatal suture	Significantly greater increase in nasal floor and maxillary base and palatal suture. (p < 0.01)
Yacout et al. (2022), [28].	Slow vs. rapid miniscrew-supported maxillary expansion	Before expansion (T1)	5 months postexpansion (T2)	-	-	5 months passive retention using same appliance	Nasal cavity width, retropalatal airway, retroglossal airway, total airway volume	Significantly greater expansion of the anterior section of the nasal cavity achieved using the slow activation protocol. (p < 0.05)
Sim et al. (2023) [29]	MARPE (pre- vs. post-expansion CBCT comparison)	Before expansion (T1)	Post-expansion (T2)	>5 months postexpansion (T3)	-	5 months passive retention using same appliance	Inferior nasal meatus (INM) volume, tortuosity ratio (TR) measurement, three dimensional (3D) nasal septum evaluation, Linear measurements the nasal septum (C point) and the lateral wall of nasal cavity	INM increased significantly at T2 and was retained at T3 after MARPE. No significant effect on nasal deviation
Yacout et al. (2021) [30]	Slow vs. rapid miniscrew- supported maxillary expansion	Before expansion (T1)	-	5 months after initial activation of appliance (T2)	-	5 months passive retention using same appliance	Nasal cavity width at premolar and molar region. (NCW-4 and NCW-6)	Significant increase in NCW-4 and NCW-6 in both groups. (p < 0.001)
Tang et al. (2021) [31]	MARME (pre- vs. post-expansion CBCT comparison)	Before expansion (T1)	Postexpansion (T2)	-	After 1 year of retention (T3)	3 months; jack screw locked. After 3 months stainless steel arms were removed but jack-screw and implants maintained in place till orthodontic treatment was completed.	Nasal floor width (NF), nasal width between the most lateral walls of nasal cavity, maxillary width parallel to the line NF and 10 mm above the line (NF10)	Significant positive correlation was observed between palatal cortical bone thickness (PCBT) and postexpansion change (T2-T1) in NF (p < 0.05). The smallest rate of decrease was noted at N-N
Al-Mansour et al. (2022) [32]	MARME (pre- vs. post-expansion CBCT comparison)	Before expansion (T0)	After 3 months (T1)	After 6 months of expansion (T2)	-	Not mentioned	Change in nasal volume, pharyngeal volume, total volume	Significant increase in nasal, pharyngeal, and total volume at T1-T2 and T0-T2
Chen et al. (2025) [33]	MARPE (pre- vs. post-expansion CBCT comparison)	Before expansion (T0)	Immediately after active expansion (T1)	-	One year post-expansion (T2)	3 months passive retention with same appliance; after 3 months molar bands removed, expansion screw and mini screws maintained for 12 months it removed 1 month before orthognathic surgery	Nasal Cavity width (NCW), Nasal floor width (NFW)	The initial NFW increase demonstrated a relapse of 0.6 ± 1.2 mm, or 11.6% of the initial expansion. (p < 0.01)

Certainty of Evidence

Inconsistency was judged as not serious for most outcome measurements, as the direction of effect was generally uniform across studies. However, for long-term stability, inconsistency was rated serious because some studies reported no significant change, some noted continued improvement, while a few observed partial relapses over time. Indirectness was judged not serious for all outcomes, as the included studies directly addressed the review question using comparable interventions and outcome measures. Imprecision was rated serious across all outcomes, as most studies had small sample sizes and wide confidence intervals.

Five of the included studies were published in non-indexed or low-impact journals with unclear peer-review processes, which increased the risk of publication bias. These studies were Alawady et al. [17], Shendy et al. [18], Sim et al. [29], Yacout et al. [30], and Al-Mansour et al. [32]. Accordingly, the certainty of evidence for related outcomes was downgraded under this domain. Overall, the certainty of evidence was graded as low to very low across all outcomes, primarily due to serious risk of bias, imprecision, and possible publication bias, despite the studies directly addressing the review question.

All measurements were reviewed for unit consistency. Some measurements were reported directly in cm³ or as percentage change and were therefore presented in their original units when conversion to mm³ was not feasible or appropriate; this was done to avoid introducing errors due to misinterpretation or unit-related errors. Implausible values were corrected based on the original study units (Table 5).

**Table 5:** GRADE evidence profile for the effects of maxillary expansion on upper airway dimensions. Risk of bias, inconsistency, indirectness, imprecision, and publication bias were judged according to the GRADE approach for each outcome. Overall certainty of evidence was rated as high, moderate, low, or very low. Long-term stability was defined as follow-up ≥12 months. RCT = randomized controlled trial; RS = retrospective study; PS = prospective study; GRADE = Grading of Recommendations Assessment, Development, and Evaluation

Outcome	Number of studies	References and study design	Risk of bias	Inconsistency	Indirectness	Imprecision	Publication bias	Certainty of evidence
Nasal cavity width	10	Bazargani et al. (2020), [19]. RCT Celenk-Koca et al. (2018), [20]. RCT Mehta et al. (2022), [21]. RS Garib et al. (2021), [24]. RS Lee et al. (2022), [25]. RS Oh et al. (2019), [27]. RS Yacout et al. (2022), [28]. RS Yacout et al. (2021), [30]. PS Tang et al. (2021), [31]. RS Chen et al. (2025), [33]. PS	Serious	Not serious	Not serious	Serious	Not serious	Low
Nasal floor width	7	Alawady et al. (2025), [17]. RCT Bazargani et al. (2020), [19]. RCT Celenk-Koca et al. (2018), [20]. RCT Lee et al. (2022), [25]. RS Oh et al. (2019), [27]. RS Tang et al. (2021), [31]. RS Chen et al. (2025), [33]. PS	Serious	Serious	Not serious	Serious	Not serious	Very low
Nasal cavity volume	3	Shendy et al. (2022), [18]. RCT Kim et al. (2018), [26]. RS Sim et al. (2023), [29]. RS	Serious	Serious	Not serious	Serious	Serious	Very low
Nasopharynx volume	2	Shendy et al. (2022), [18]. RCT Kim et al. (2018), [26]. RS	Serious	Serious	Not serious	Serious	Serious	Very low
Total volume	4	Shendy et al. (2022), [18]. RCT Kim et al. (2018), [26]. RS Yacout et al. (2022), [28]. RS Al-Mansour et al. (2022), [32]. PS	Serious	Serious	Not serious	Serious	Serious	Very low
Alar base width	2	Mehta et al. (2022), [21]. RS Empson et al. (2023), [22]. RS	Some concerns	Serious	Not serious	Serious	Not serious	Moderate
Long term stability ≥12 months	7	Bazargani et al. (2020), [19]. RCT Mehta et al. (2022), [21]. RS Empson et al. (2023), [22]. RS Ahmida et al. (2023), [23]. RS Kim et al. (2018), [26]. RS Tang et al. (2021), [31]. RS Chen et al. (2025), [33]. PS	Serious	Serious	Not serious	Serious	Not serious	Very low

To facilitate comparison of measurement protocols, the three-dimensional imaging modality, analysis software, reference standards, and head orientation methods adopted in each study are summarized in Table 6.

**Table 6:** Three-dimensional imaging protocols, analysis software, reference standards, and head orientation used in the included studies. *: MSP, APP, and vertical coronal planes were used as reference planes for image reorientation in Mimics software. CBCT = cone-beam CT; FH plane = Frankfort horizontal plane; MSP = midsagittal plane; APP = anteroposterior plane; ANS = anterior nasal spine

Study	3D imaging	Analysis software	Reference standard	Head orientation
Alawady et al. (2025) [17]	CBCT	Mimics software	MSP, APP, V coronal plane*	Not specified
Shendy et al. (2022) [18]	CBCT	Romexis software	FH plane	Standardized tongue position
Bazargani et al. (2020) [19]	CBCT	OsiriX Imaging Software	REFORMATTING = axial view = midpalatal suture; sagittal view = hard palate; coronal view= Crista galli	Not specified
Celenk-Koca et al. (2018) [20]	CBCT	OsiriX Imaging Software	Perpendicular to MPS	Not specified
Mehta et al. (2022) [21]	CBCT	Dolphin imaging software	Anterior cranial base	Not specified
Empson et al. (2023) [22]	CBCT	Dolphin imaging software	Anterior cranial base	Not specified
Ahmida et al. (2023) [23]	CBCT	Dolphin imaging software	FH plane	Not specified
Garib et al. (2021) [24]	CBCT	Nemoscan software	Palatial plane parallel to horizontal plane, orbital plane (frontal view) parallel to horizontal plane, anterior and posterior nasal spine with vertical plane	Standardized
Lee et al. (2022) [25]	CBCT	OnDemand3D	FH plane	Not specified
Kim et al. (2018) [26]	CBCT	OnDemand3D	FH plane	Orbital floor parallel to the ground
Oh et al. (2019) [27]	CBCT	InVivo 6.0 software	FH plane, Midsagittal plane, Frontal plane	Not specified
Yacout et al. (2022) [28]	CBCT	OnDemand3D	FH plane	Seated upright; head supported with headrest
Sim et al. (2023) [29]	CBCT	OnDemand3D	FH plane	Seated upright; head supported with headrest
Yacout et al. (2021) [30]	CBCT	OnDemand3D	Not specified	Not specified
Tang et al. (2021), [31].	CBCT	Dolphin imaging software	The images were reoriented along the palatal suture, tangent to the nasal floor and parallel to the palatal plane.	Not specified
Al-Mansour et al. (2022), [32].	CBCT	Mimics software	Not specified	Not specified
Chen et al. (2025), [33].	CBCT	Maxilim software	Horizontal plane passing through ANS point	Not specified

Detailed quantitative changes in nasal cavity width, nasal floor width, airway volumes, alar base width, and long-term stability following maxillary expansion are summarized in Table 7.

**Table 7:** Quantitative changes in nasal cavity width, nasal floor width, airway volumes, alar base width, and long-term stability following maxillary expansion. Mean changes are reported as mm, mm³, or percentage change, as presented in the original studies. Long-term stability was defined as follow-up ≥12 months. *: Some measurements were reported in cm³ or as percentage change and were presented in their original units when conversion to mm³ was not feasible or appropriate. NCW = nasal cavity width; N1, N2 = nasal floor and nasal cavity width measurements at different vertical positions; NCV = nasal cavity volume; NV = nasal volume; NF = nasal floor; NFW = nasal floor width; PNCW = posterior nasal cavity width; ANCW = anterior nasal cavity width; ABW = alar base width; NC = nasal cavity; INM = inferior nasal meatus; ANS = anterior nasal spine; PNS = posterior nasal spine; SME = slow maxillary expansion; RME = rapid maxillary expansion; RS = retrospective study; PS = prospective study

Outcome	Participants	Study	Mean change (mm)	Follow-up duration	Effect direction/Magnitude
Nasal cavity width	430	Bazargani et al. (2020) [19]	Position 0 N2 = 2.6 mm; position 1 N2 = 2.3 mm; position 2 N2 = 2.6 mm; position 3 N2 = 1.9 mm	1 year	Significant increase postexpansion, no significant change 1 year postexpansion
		Celenk-Koca et al. (2018) [20]	NCWP = 2.8 ± 1.8 mm; NCWM = 2.9 ± 1.7 mm	6 months	Significant increase at 6 months postexpansion CBCT
		Mehta et al. (2022) [21]	ANCW = 1.67 mm; PNCW = 1.66 mm	2 years 8 months	Significant increase postexpansion, with additional increase observed posttreatment
		Garib et al. (2021) [24]	NCW = 2.26 ± 1.17 mm	11 months	Significant increase at 11 months postexpansion CBCT
		Lee et al. (2022) [25]	LNW: R = 1.11 ± 0.60 mm; L = 1.15 ± 0.70 mm	6 months	Significant increase, greater increase in dimension on the right side at T2 and on the left side at T3
		Oh et al. (2019) [27]	w_LN = 1.38 ± 1.6 mm; w_IN = 2.26 ± 1.25 mm	6 months	Significant increase at 6 months postexpansion CBCT
		Yacout et al. (2022) [28]	SME T2-T1: Anterior = 4.44 ± 1.98 mm; middle = 3.00 ± 1.05 mm; posterior = 1.99 ± 1.04 mm. RME T2-T1: Anterior = 1.08 ± 1.86 mm; middle = 2.35 ± 1.11 mm posterior = 2.84 ± 1.31 mm	5 months	Significant increase, greater increase in anterior section of nasal cavity with SME
		Yacout et al. (2021) [30]	NCW-4 S = 2.95 ± 0.046 mm; R = 2.54 ± 0.83 mm; NCW-6 S = 2.62 ± 0.92 mm; R = 2.57 ± 0.87 mm	5 months	Significant increase in NCW in both groups at 5 months. Greater increase in NC at premolar and molar region in SME, but difference not significant between the groups
		Tang et al. (2021) [31]	N-N T2-T0: 2.12 ± 1.08 mm	1 year	Significant increase at T1, slight relapse at T2 but statistically significant. Indicating increase in nasal cavity width is relatively irreversible
		Chen et al. (2025) [33]	NCW T2-T0: 2.3 ± 1.6 mm	1 year	Significant increase at T1 and T2, significant relapse at T2 compared with T1
Nasal floor width	304	Alawady et al. (2025) [17]	Group 1: RT. ANS = 2.01 ± 0.77 mm; LT. ANS = 1.94 ± 0.51 mm; RT. PNS = 1.50 ± 0.56 mm; LT. PNS = 1.52. ± 0.54 mm; Group 2: RT. ANS = 1.96 ± 0.74 mm; LT. ANS = 1.84 ± 0.82 mm; RT. PNS = 1.63 ± 0.60 mm; LT. PNS = 1.54 ± 0.58 mm	6 months	Significant increase at 6 months irrespective of MOP protocol
		Bazargani et al. (2020) [19]	Position 0 N1 = 1.9 mm; position 1 N1 = 2.3 mm; position 2 N1 = 2.4 mm; position 3 N1 = 0.8 mm	1 year	Significant increase post expansion, no significant change 1 year postexpansion
		Lee et al. (2022) [25]	Right = -0.83 ± 1.16 mm; left = 0.95 ± 0.93 mm	6 months	Significant increase at T2 and T3. Statistically significant relapse at T3 compared to T2
		Tang et al. (2021) [31]	NF = 1.98 ± 1.29 mm	1 year	Significant increase at T1 and T2, with slight relapse at T2 compared to T1
		Chen et al. (2025) [33]	NFW = 3.0 ± 1.4 mm	1 year	Significant increase at T1 and T2, significant relapse at T2 compared with T1
Nasal cavity volume	73	Shendy et al. (2022) [18]	Percentage change NCV = 22.73%	6 months	Significant increase at 6 months
		Kim et al. (2018) [26]	1710.2 ± 881.6 mm³	1 year	Significant increase at T1, significant additional increase at T2
		Sim et al. (2023) [29]	INM T0-T2: 443.58 ± 293.36 mm³	>5 months	Significant increase at T1 and T2. Slight relapse at T2 compared to T1 but not statistically significant
		Al-Mansour et al. (2022) [32]	NV T0-T2: 20.81 ± 3.23 mm³	6 months	Significant increase at T1, with additional statistically significant increase at T2
Nasopharynx volume	44	Shendy et al. (2022) [18]	Percent change: 19.75%	6 months	Significant increase at 6 months postexpansion
		Kim et al. (2018) [26]	942.4 ± 821.0 mm³	1 year	Increase noted at T1, with statistically significant improvement only at 1 year postexpansion
Total volume	76	Shendy et al. (2022) [18]	Percent change: 18.76%	6 months	Significant increase at 6 months postexpansion
		Kim et al. (2018) [26]. RS	2652.6 ± 221.2 mm³	1 year	Increase noted at T1, with statistically significant improvement only at 1 year postexpansion
		Yacout et al. (2022) [28] RS	SME = 175.76 mm³; RME = 523.19 mm³	5 months	Non-significant increase observed in both SME and RME groups
		Al-Mansour et al. (2022) [32] PS	4.89 ± 1.76 cm³	6 months	Statistically significant increase at Ṭ1-T2 and T0-T2
Alar base width	60	Mehta et al. (2022) [21] RS	ABW = 0.31 mm	2 years 8 months	Significant increase postexpansion, with additional increase observed post-treatment
		Empson et al. (2023) [22]. RS	Right alar base (T2-T1) x = -0.38 mm; y = -1.57 mm; z = 0.05 mm. Left alar base x = 0.35 mm; y = -0.21 mm; z = -0.33 mm	2 years 8 months	Significant changes in the short term, no changes observed in the long term
Long-term stability ≥12 months	190	Bazargani et al. (2020) [19] RCT	N1: T2-T1 = 1.9 mm; N2: T2-T1 = 2.6 mm	1 year	Significant increase post expansion, no significant change 1 year postexpansion
		Mehta et al. (2022) [21]	PNCW: T2-T1 = 6.78% (p < 0.001*); ANCW: T2-T1 = 7.99% (p < 0.001*)	2 years 8 months	Significant increase postexpansion, with additional increase observed post-treatment
		Empson et al. (2023) [22]	Right alar base (T3-T1) x = -0.39 mm; y = -2.93 mm; z = 0.87 mm. Left alar base x = 0.57 mm; y = -0.51 mm; z = -0.03 mm	2 years 8 months	Significant changes in the short term, no changes observed in the long term
		Ahmida et al. (2023) [23]	MPS molar: T3-T1 = 0.37 mm (p< 0.001*)	2 years 8 months	Significant increase at T2 and T3 with in long term
		Kim et al. (2018) [26]	NC = 1710.2 ± 881.6; nasopharynx = 942.4 ± 821.0 mm³; total volume = 2,652.6 ± 221.2 mm³	1 year	Increase noted at T1, with statistically significant improvement only at 1 year postexpansion
		Tang et al. (2021) [31]	N-N: 5.76% relapse	1 year	Significant increase with some amount of relapse long term
		Chen et al. (2025) [33]	NFW: 0.6 ± 1.2 mm, 11.6% relapse	1 year	Initial increase followed by relapse

The types of maxillary expansion appliances, miniscrew configuration, activation protocols, and criteria for terminating activation in the included studies are summarized in Table 8.

**Table 8:** Types of maxillary expansion appliances, miniscrew configuration, activation protocols, and termination criteria in the included studies. MARPE = miniscrew-assisted rapid palatal expansion; MARME = miniscrew-assisted rapid maxillary expansion; MSE = maxillary skeletal expander; MSME = miniscrew-supported maxillary expander; RME = rapid maxillary expansion; SME = slow maxillary expansion; TBB RME = tooth–bone-borne rapid maxillary expansion; MS RME = miniscrew-supported rapid maxillary expansion; BAME = bone-anchored maxillary expander; D-MED = Dutch Maxillary Expansion Device; CBCT = cone-beam CT; MD = midline diastema

Study	Type of MARPE Used	No. of Implants	Implant Dimensions	Activation Protocol	Termination of Activation
Alawady et al. (2025) [17]	MSE	4	1.8 mm × 11 mm	Two turns in the morning, two turns in the evening (0.13mm expansion each turn)	When palatal cusp of maxillary molar occluded with buccal cusp of mandibular permanent first molar
Shendy et al. (2022) [18]	MSE	4	1.8 mm × 11 mm	Two quarter turns per day	16 days
Bazargani et al. (2020) [19]	(TBB) RME	2	1.7 mm × 8mm	Two quarter turns per day	When palatal cusp of maxillary molar occluded with buccal cusp of mandibular permanent first molar
Celenk-Koca et al. (2018) [20]	MS RME	4	1.8 mm × 9 mm	Two quarter turns per day	When palatal cusp of maxillary molar occluded with buccal cusp of mandibular permanent first molar
Mehta et al. (2022) [21]	MARPE	2	1.5 mm × 12 mm	Two quarter turns per day	Until posterior overbite correction
Empson et al. (2023) [22]	MARPE	2	1.5 mm × 12 mm	Two quarter turns per day	Until posterior overbite correction
Ahmida et al. (2023) [23]	MARPE	2	1.5 mm × 12 mm	Two quarter turns per day	Until posterior overbite correction
Garib et al. (2021) [24]	MSE	2	1.8 mm × 7 mm	Two quarter turns per day	14 days
Lee et al. (2022) [25]	MSE	4	1.5 mm × 11 mm	One turn per day for early teens (<15); 2 turns per day for late teens (>=15); 4 turns per day adults (>20), after MD two turns per day regardless of age till proper expansion achieved	Until proper expansion achieved
Kim et al. (2018) [26]	MARME	4	1.8 mm × 7 mm	Once a day (0.2 mm/turn)	28 days
Oh et al. (2019) [27]	BAME	2+2	2 onplants - 3 mm × 8 mm; 2 mini-screws - 1.5 mm × 12 mm	Two quarter turns per day	Till 20% over correction of posterior cross bite
Yacout et al. (2022) [28]	MSE	4	1.6 mm × 10 mm	SME group = once every other day (0.2 mm); RME group = twice daily (0.4 mm)	Until transverse discrepancy corrected
Sim et al. (2023) [29]	MSE	4	Length - 11 mm	1 or 2 turns per day (a types of MSE = 0.8 mm for 4 turns, 0.8 mm for 6 turns)	2.3 (±2.1) months
Alawady et al. (2025) [17]	MSME	4	1.6 mm × 10 mm	SME group = once every other day (0.2 mm); RME group = twice daily (0.4 mm)	Until transverse discrepancy corrected
Shendy et al. (2022) [18]	MARME	4	1.5 mm × 11 mm	One turn per day (0.13 mm per turn)	40–60 turns
Bazargani et al. (2020) [19]	MSE	4	Not specified	Two turns per day till diastemma then one turn per day	Not specified
Celenk-Koca et al. (2018) [20]	Dutch Maxilllary Expansion Device (D-MED)	4	Appropriate length selected according to thickness of palatal bone determined by CBCT	Once a day	Until required amount of expansion achieved

Discussion

The present systematic review evaluated the impact of MARPE and other bone-borne expanders on airway dimensions using CBCT-based assessments. Transverse maxillary deficiency is a prevalent condition, and timely intervention in nearly 80% of cases is essential to prevent functional complications associated with occlusion and airway obstruction [34,35]. MARPE achieves maxillary separation at the midpalatal suture and produces primarily skeletal changes. The suture typically opens in a V-shaped manner, wider anteriorly than posteriorly, due to resistance from the pterygoid plates of the sphenoid bone [2,36]. Because of the close anatomical relationship between the maxilla and the nasal cavity, maxillary expansion secondarily widens the nasal floor [32].

Lagravère et al. [37] reported that only about 25% of expansion achieved through RME is skeletal, while the remaining 75% is alveolar [37]. Although several included studies found comparatively greater skeletal effects with bone-borne expanders, definitive conclusions regarding functional impact cannot be drawn and would require dedicated functional testing [18-24,27]. Alawady et al. [17] investigated whether adding MOPs to MSE could enhance expansion by comparing two MOP approaches, one limited to the MPS and another combining MPS and buccal perforations [17]. Both achieved comparable skeletal expansion, with no statistically significant difference between them. This suggests that while MOPs may facilitate bone remodeling by reducing sutural resistance, their additional benefit beyond standard MSE remains limited. These findings are in agreement with Suzuki et al., who proposed the use of corticopuncture to stimulate the regional acceleratory phenomenon, enhancing local bone turnover and reducing cortical resistance [38].

Shendy et al. reported that MSE produced significantly greater increases in total airway, nasopharyngeal, retropalatal, retroglossal, and nasal cavity volumes compared to hybrid or conventional Hyrax expanders [18]. Not only that, the bone-borne design of MSE facilitated a more uniform skeletal opening, leading to superior volumetric outcomes. Bazargani et al. also found greater nasal cavity widening in the TBB expander group compared to the tooth-borne group, with stable results one year after expansion [19]. They concluded that tooth-borne RME may suffice for younger patients without airway compromise, whereas TBB RME offers enhanced skeletal and airway effects in patients with nasal obstruction. Similarly, Celenk-Koca et al. observed that bone-borne expanders produced greater nasal cavity expansion, particularly in the molar region, than conventional appliances [20].

Mehta et al. [21] demonstrated a significant reduction in NSDA and a greater posterior nasal cavity width increase using MARPE [21]. Empson et al. [22] observed that MARPE produced no long-term adverse soft tissue changes, while Ahmida et al. [23] found significant midpalatal suture widening without detrimental effects on adjacent circummaxillary sutures [22,23]. Lee et al. [25] further reported that nasal septal deviation was alleviated following MSE, with both short- and long-term improvements in nasal septal length and lateral nasal wall width [25]. Kim et al. [26] discovered significant increases in nasal cavity and nasopharyngeal volumes after expansion, with additional gains noted at one-year follow-up [26]. Furthermore, the magnitude of increase in the nasal cavity exceeded that of the nasopharynx, indicating that MARPE’s skeletal effects are most pronounced in the anterior airway region. These findings are consistent with Cantarella et al. [39], who reported approximately 4.3 mm of skeletal expansion with MSE, alongside measurable improvements in nasal volume and morphology [39].

While most studies demonstrated favorable outcomes, some variability exists in the improvement of airway dimensions. This heterogeneity may be attributed to differences in patient age, skeletal maturity levels, activation protocols, appliance designs, and CBCT segmentation methods used to quantify airway changes. Additionally, due to the visibility of the appliances, most randomized trials did not perform blinding of outcome assessment; this introduced some risk of bias [17-24].

Both slow and rapid activation protocols resulted in significant lateral expansion of the nasal cavity, complementing maxillary skeletal widening [28]. Earlier studies using both slow and rapid activation reported similar findings. Yacout et al. noted that slow expansion produced a significantly larger increase in anterior nasal cavity width than rapid activation. Accordingly, they emphasized its importance in improving airflow as the anterior nasal segment contributes the greatest resistance to nasal breathing [28]. The effect of miniscrew-supported expansion on upper airway volume, however, was not significant regardless of activation rate. This is consistent with Kabalan et al. [40] and Li et al. [41], who found no substantial change in retropalatal or retroglossal airway volume following expansion [40,41]. The lack of significant change may be due to the remoteness of the oropharyngeal airway from the point of force application, as Ghoneima et al. previously noted that RME primarily influences anterior craniofacial sutures [42].

Mehta et al. also reported a significant increase in oropharyngeal airway volume, though their definition did not include the retroglossal segment [21]. Such differences in landmark definitions, alongside differences in CBCT protocols, appliance designs, and timing of postexpansion assessments, may contribute to variations in results across different studies. Tang et al. highlighted the importance of maintaining expansion with a rigid device, as the opened palatal suture tends to relapse under periosteal and muscular tension [31]. In their study, the jackscrew and four mini-implants were retained as a MARPE retainer for more than one year, yielding favorable stability [31]. Thicker palatal cortical bone was associated with greater postexpansion stability, suggesting that bone rigidity influences relapse tendency [31]. Similarly, Chen et al. reported a skeletal relapse rate of only 11.6%, which was statistically but not clinically significant. Furthermore, the skeletal relapse at the nasal floor was less than at the dental level, producing a characteristic V-shaped expansion pattern in the coronal plane [33].

From a clinical perspective, the observed improvements in nasal width, airway volume, and skeletal dimensions following MARPE support its effectiveness in managing maxillary constriction. However, the effects of these improvements on airway function should be interpreted cautiously, as functional outcomes were not included in this review. In addition, although many short-term outcomes showed increases, some parameters did not reach statistical significance. Long-term findings (≥12 months) were similarly variable, with studies reporting stable outcomes, no change, or partial relapse.

Limitations

Despite promising outcomes, the current evidence is limited by small sample sizes, methodological heterogeneity, and variable follow-up durations. The broad participant age range of 8-33 years, with limited distinction between growing and skeletally mature individuals, may further introduce confounding from natural growth and should be considered when interpreting the airway changes. The use of MARPE, MSE, hybrid, and other bone-borne expanders introduces biomechanical and clinical variability. While these appliances share the principle of skeletal anchorage, differences in design and force distribution could lead to varying airway outcomes; therefore, findings resulting from evaluating them as a single group should be interpreted with caution. In addition, the review protocol was not prospectively registered in a database such as PROSPERO, which may be considered a methodological limitation.

To strengthen the evidence base, future studies should include standardized CBCT imaging protocols along with long-term functional assessments, such as rhinomanometry or airflow testing, which are needed to confirm the durability and clinical relevance of these findings.

## Conclusions

Within the limits of low to very low certainty evidence, CBCT-based studies indicate that MARPE and other bone-borne expanders produce consistent skeletal widening and modest increases in nasal and nasopharyngeal dimensions, with partial maintenance of these changes over follow-up periods of up to one year or more. However, the magnitude of these dimensional gains varies between studies, and their true functional impact on breathing remains unclear. Therefore, MARPE can be considered a promising, less invasive option for managing transverse maxillary deficiency in selected patients, but its effects on airway performance should not yet be regarded as predictable. Future high-quality, prospective studies combining standardized CBCT protocols with objective functional assessments are required to determine the clinical significance of these radiographic changes.
